# Biocompatibility of common implantable sensor materials in a tumor xenograft model

**DOI:** 10.1002/jbm.b.34254

**Published:** 2018-10-27

**Authors:** Mark E. Gray, James Meehan, Ewen O. Blair, Carol Ward, Simon P. Langdon, Linda R. Morrison, Jamie R. K. Marland, Andreas Tsiamis, Ian H. Kunkler, Alan Murray, David Argyle

**Affiliations:** ^1^ The Royal (Dick) School of Veterinary Studies and Roslin Institute University of Edinburgh Edinburgh EH25 9RG UK; ^2^ Cancer Research UK Edinburgh Centre and Division of Pathology Laboratories, Institute of Genetics and Molecular Medicine University of Edinburgh Edinburgh EH4 2XU UK; ^3^ Institute of Sensors, Signals and Systems, School of Engineering and Physical Sciences Heriot‐Watt University Edinburgh EH14 4AS UK; ^4^ School of Engineering, Faraday Building Edinburgh EH9 3JL UK

**Keywords:** biocompatibility, tumor xenograft model, tumor microenvironment, innate immune response, foreign body response, implantable biosensor

## Abstract

Real‐time monitoring of tumor microenvironment parameters using an implanted biosensor could provide valuable information on the dynamic nature of a tumor's biology and its response to treatment. However, following implantation biosensors may lose functionality due to biofouling caused by the foreign body response (FBR). This study developed a novel tumor xenograft model to evaluate the potential of six biomaterials (silicon dioxide, silicon nitride, Parylene‐C, Nafion, biocompatible EPOTEK epoxy resin, and platinum) to trigger a FBR when implanted into a solid tumor. Biomaterials were chosen based on their use in the construction of a novel biosensor, designed to measure spatial and temporal changes in intra‐tumoral O_2_, and pH. None of the biomaterials had any detrimental effect on tumor growth or body weight of the murine host. Immunohistochemistry showed no significant changes in tumor necrosis, hypoxic cell number, proliferation, apoptosis, immune cell infiltration, or collagen deposition. The absence of biofouling supports the use of these materials in biosensors; future investigations in preclinical cancer models are required, with a view to eventual applications in humans. To our knowledge this is the first documented investigation of the effects of modern biomaterials, used in the production of implantable sensors, on tumor tissue after implantation. © 2018 The Authors. *Journal of Biomedical Materials Research Part B: Applied Biomaterials* published by Wiley Periodicals, Inc. J Biomed Mater Res Part B, 2018. © 2018 Wiley Periodicals, Inc. J Biomed Mater Res Part B: Appl Biomater 107B: 1620–1633, 2019.

## INTRODUCTION

Cancer is a leading cause of mortality, resulting in personal, economic and social burdens in developing and developed countries alike.^1^ Novel diagnostic, monitoring, and treatment regimens are required if long‐term survival rates are to be improved. The metabolic processes within solid tumors differ from those in normal tissues. Areas within tumors can be deprived of O_2_, glucose and energy, while experiencing extracellular acidosis, high lactate levels and interstitial hypertension, caused by abnormal tumor vasculature.^2^ Monitoring the tumor microenvironment (TME) for cancer biomarkers, metabolites, pH, and O_2_ could provide data to target hypoxic areas more effectively by radiation and anti‐cancer drugs; data could also be used to monitor the response of a tumor to treatment, detect residual or recurrent disease and help understand the biological events that drive the metastatic process.^3^, ^4^

Identification of hypoxic areas within solid tumors is clinically important as hypoxic cancer cells are more resistant to chemo/radiotherapy, with increased invasive and metastatic potential. Advanced radiotherapy (RT) delivery systems allow dose distribution to be applied with great accuracy to tumors while sparing normal tissues.^5^ Conventional curative RT schedules for solid tumors (e.g., lung, head and neck, breast) deliver the same dose distribution to the whole tumor based on a baseline radiation distribution plan in a series of fractions over several weeks. Continuous intra‐tumoral monitoring of oxygenation could create a changing map of the distribution of hypoxia. This would enable dose distribution to be modulated, optimized, and individualized on a daily basis to improve tumor response. Additional dosage could also be delivered to hypoxic areas. An implantable biosensor taking intra‐tumoral real‐time O_2_ readings would provide information at the time of treatment, monitoring spatial, and temporal changes thus overcoming the limitations associated with current technologies to measure tumor oxygenation status. The Implantable Microsystems for Personalised Anti‐Cancer Therapy (IMPACT) project (www.impact.eng.ed.ac.uk) aims to produce such a device, allowing RT to be delivered at the most effective location and time by targeting these hypoxic regions.^4^

Implantable peri‐tumoral or intra‐tumoral devices could also be developed to release chemotherapeutic drugs directly within the TME, increasing the drugs therapeutic potential while reducing the severity of systemic side effects.^6^ Implantable devices have also been used in radiation oncology; an implantable dosimeter has been developed to verify the radiation dose received by the target volume for each fraction the patient receives.^3^ This dosimeter has undergone clinical testing and received FDA approval for use in breast and prostate cancer and can allow a radiation oncologist to optimize radiation treatment on an individual basis.

For implantable devices to gain clinical approval, their biocompatibility, incorporating both bio‐functionality and biosafety, must be investigated.^7^, ^8^, ^9^, ^10^ Unfortunately, the biocompatibility of implantable devices remains a challenge as biosensors typically lose functionality over time; this detrimental effect is largely due to biofouling (non‐specific cell/protein absorption) that occurs locally around the device, resulting in a tissue reaction known as the foreign body response (FBR).^11^, ^12^, ^13^ Biocompatibility can also be viewed as a characteristic of a system and not of a specific material; individual materials may therefore affect different biological systems in different ways.^13^, ^14^, ^15^ The focus of the FBR currently relates to materials that are implanted into normal tissue, with numerous *in vivo* models available for biocompatibility testing.^16^, ^17^, ^18^, ^19^, ^20^ If medical devices are developed for implantation within or near tumor tissue, then the FBR must also be evaluated within these diseased tissues, rather than relying on previously published FBR data from implantation within healthy tissue alone. The purpose of this study was to develop a novel murine model where biomaterials, that were under consideration for use in the IMPACT biosensor, could be safely and reproducibly implanted into human cancer cell xenografts. Novel methodology was developed to process tumors while biomaterials were still present and to section them to identify the implant site. The effects of the biomaterials were investigated through changes in body weights and mean tumor volumes, while immunohistochemistry was used to assess necrosis, proliferation, apoptosis and hypoxic markers, as well as innate immune responses and fibrosis within the tumor. Literature searches indicate that this is the first report of the interaction of modern biomaterials used in implantable biosensor technology with a TME.

## MATERIALS AND METHODS

### Biomaterial fabrication

The IMPACT biosensor consists of a microfabricated silicon chip insulated in biocompatible resin. The outward‐facing materials of the biosensor were selected for testing; these were: silicon dioxide (SiO_2_), silicon nitride (Si_3_N_4_), Parylene‐C, Nafion, OG116‐31 resin (Epoxy Technology), and platinum (Pt). Materials were prepared in the Scottish Microelectronics Centre Class 10 cleanroom facility (Kings Buildings, University of Edinburgh) and comprised of 3–7 mm long pieces of titanium (Ti) wire, diameter 0.4 mm, coated with the material to be tested. Copper (Cu) wire (Sigma Aldrich, 99.999% purity) was used for positive control samples.

### Biomaterial manufacture

For all coated biomaterials Ti wire was first cleaned in isopropyl alcohol at 50°C with ultrasonic agitation for 15 min, followed by the same treatment in deionized water, then dried using an N_2_ gun; Cu wire was also cleaned using the same protocol. Parylene‐C samples were produced using a vapor deposition system SCS (Speciality Coating Systems 2010 Labcoater) ensuring a conformal coating of 5 μm of Parylene‐C. SiO_2_ and Si_3_N_4_ samples were prepared using Plasma Enhanced Chemical Vapour Deposition (PECVD); a 1 μm layer of each material was deposited. Pt samples were produced using electron‐beam evaporation in an ANS Cluster tool which deposited a 50 nm thick Pt film onto the wire. Nafion samples were created by dipping Ti wire in a solution of 5% by weight Nafion in lower aliphatic alcohols and water, before air curing for 5 min; the process was repeated five times before curing at 120°C for 1 h.^21^ Resin samples were produced by dip coating the Ti wire in OG116‐31 resin then curing for 800 s under ultraviolet light. After completion of each coating process the wires were optically inspected to ensure uniformity.

### Generation of MDA‐MB‐231 xenograft tumors

Murine studies were undertaken under a UK Home Office Project Licence. The study was performed in accordance with the Animals (Scientific Procedures) Act 1986, which was approved by the University of Edinburgh Animal Ethics Committee. Recommended guidelines for welfare and use of animals in research were followed. CD‐1 immunodeficient female nude mice (Charles River Laboratories, Tranent, UK) of at least 8 weeks of age were allowed a period of adaptation in a sterile, pathogen‐free environment, with *ad libitum* access to food and water. Mice were housed in individually ventilated cages in a barrier environment.

MDA‐MB‐231 human breast tumor cells (originally obtained from ATCC) were grown routinely for the generation of stock xenografts. Approximately 2 × 10^9^ MDA‐MB‐231 cells were re‐suspended in 1 mL of serum‐free DMEM (Gibco® Life Technologies, Invitrogen, UK), with 0.1 mL of this cell suspension injected bilaterally into subcutaneous tissue of the flanks of five mice. Once stock tumors had grown sufficiently (6 weeks) they were harvested and sectioned into 1–2 mm long fragments before implantation into experimental mice under local anesthetic (ethyl chloride) using a 12 G trocar. Each mouse received two tumor fragments injected bilaterally into subcutaneous flank tissue.

### Biomaterial implantation

Treatment groups consisted of xenograft tumors implanted with biomaterial, controls (xenograft tumors that received a needle tract (NT) injury) and an untreated group. Bilateral tumor generation allowed the total number of mice required to be reduced as each mouse acted as its own control; one tumor received biomaterial implantation or NT injury while the contralateral tumor was left untreated. Nafion coated wires were sterilized by routine autoclaving; all other biomaterials were sterilized in 100% ethanol for 10 min, rinsed in deionized water and stored in penicillin and streptomycin. All wires were washed in sterile distilled water immediately before implantation.

Mice underwent general anesthesia using isoflurane gaseous anesthesia for implantation (defined as day 0). Skin was aseptically prepared using chlorohexidine solution. For biomaterial implantation, a 21 G needle was used to penetrate the skin overlying the tumor but did not enter the tumor parenchyma itself. This entry point was located at the caudal aspect of the tumor so introduction of the biomaterial would be along the long axis. Biomaterial was then introduced into the tumor through the pre‐prepared entry point. Tissue adhesive (Vetbond™, 3M) was applied to the skin. For NT control tumors, a 21 G needle was used to penetrate the skin then advanced into the tumor tissue itself. A minimum of five mice were used in each treatment group. Day 0 mice were euthanized immediately after anesthesia, with the remaining mice monitored up to 7 days post‐implantation. Mice were assessed for signs of ill health with body weights, body condition score, and tumor size measured three times a week. Vernier calipers were used to measure tumor size. Tumor volume was calculated as π/6 × width^2^ × length. Relative tumor volume was calculated by dividing the tumor volume on each day by its volume on day 0.

### Immunohistochemistry

**Figure 1:**
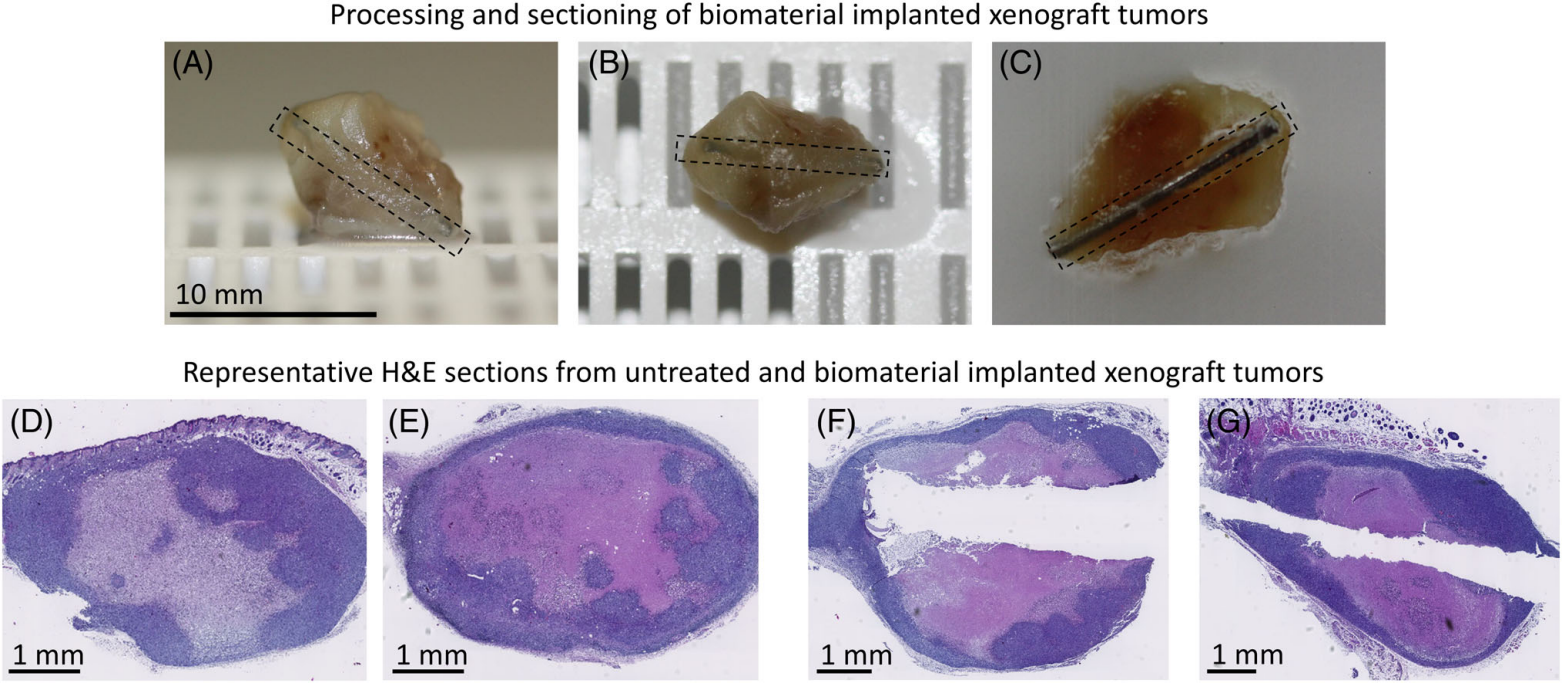
Photographs depicting xenograft tumor processing and sectioning, along with representative H&E stained slides. (A–C) Photographs depicting the position of the biomaterial within a xenograft tumor following harvesting and processing for IHC. The dashed box is outlining an OG116‐31 resin biomaterial wire. To identify the implant site, sectioning of the tissue block continued until the tip of the biomaterial was found. If the biomaterial was approximately flush with the cut surface the wire was removed and sectioning continued; however, if the direction of the wire was further into the tumor, the paraffin was then melted, and the wire carefully removed. The tumor tissue was trimmed from its sectioned edge as parallel as possible to the path of the wire tract before being re‐embedded in paraffin. Once set, sectioning continued through the block. (D–G) Representative H&E stained sections from untreated and biomaterial implanted xenograft tumors harvested at day 7. (D, E) Untreated, (F) Nafion implanted, (G) SiO_2_ implanted.

Microtome sections of 4 μm were placed on SuperFrost® Plus glass slides (Thermo Scientific™, UK) and dried overnight at 37°C. Sections for IHC underwent a routine IHC protocol and counterstaining in hematoxylin^22^ (Table 1 provides details of all antibodies used in the study). Additional sections were also routinely stained with hematoxylin and eosin (H&E) or Masson's Trichrome (TCS Biosciences Ltd, UK). All IHC slides were scanned using the NanoZoomer ER slide scanner (Hamamatsu Photonics, UK) and viewed using NanoZoomer Digital Pathology software (Figure 1).

**Table 1:** Antibodies Used in This Study

Primary antibody target antigen	Histological marker/cited publications	Antibody details and antigen retrieval solution	Manufacturer	Dilution
Anti‐ki67	Proliferation^23^, ^24^, ^25^, ^26^	Monoclonal rabbit Sodium citrate	Abcam; ab92742	1:1000
Anti‐carbonic anhydrase IX	Hypoxia^22^, ^27^, ^28^	Polyclonal rabbit Sodium citrate	Abcam: ab15086	1:750
Anti‐cleaved caspase‐3	Apoptosis^29^, ^30^, ^31^, ^32^	Polyclonal rabbit Sodium citrate	Cell Signalling Technology; 9661	1:150
Anti‐Ly‐6C/‐6G	Neutrophil^33^, ^34^, ^35^, ^36^	Monoclonal Rat EDTA	Abcam; ab25377	1:50
Anti‐F4/80	Macrophage^37^, ^38^, ^39^, ^40^	Monoclonal Rat Enzymatic	Biolegend; 123101	1:100
Anti‐ER‐TR7	Fibroblast^41^, ^42^, ^43^, ^44^	Monoclonal Rat Enzymatic	Novus Biologicals; NB100‐64932	1:50

### Immunohistochemical analysis

Image analysis software QuPath version 0.1.2 (Queen's University, Belfast) was used to analyze target protein expression and percentage area tumor necrosis. The percentage area of collagen was assessed with a color deconvolution macro developed by L. Murphy (IGMM, University of Edinburgh, UK) using Image J (NIH, Bethesda, MD). All extraneous tissue such as subcutaneous fat and skin was excluded from image analysis. The staining pattern of each antibody was verified by a board‐certified veterinary pathologist (L.R. Morrison, The Royal (Dick) School of Veterinary Studies, Edinburgh).

### Statistical analysis

Data was analyzed with parametric tests; one‐way ANOVA with Tukey's multiple comparisons was used to test for differences between more than two groups, and unpaired (two tailed) *t* test was used to test for differences between two groups. *p* values <0.05 were deemed statistically significant. Data expressed as mean ± SEM.

## RESULTS

### The effects of biomaterial implantation on mice body weights and tumor volumes

**Figure 2:**
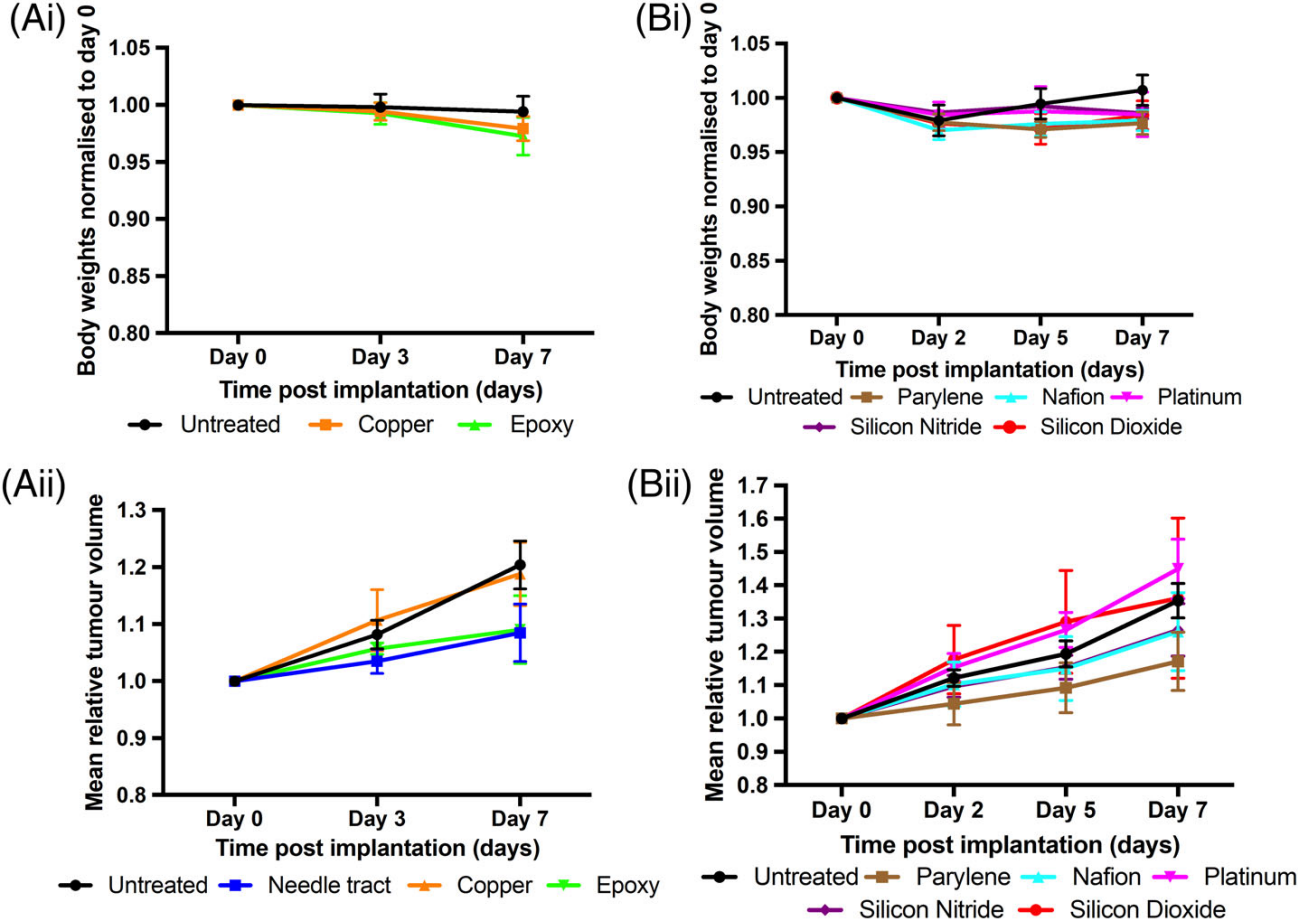
The effects of different biomaterials on mice body weights and tumor volumes. (Ai, Bi) Changes in mice body weights for untreated xenograft tumors and Cu, OG116‐31 resin, Parylene‐C, Nafion, Pt, Si_3_N_4_, and SiO_2_ implanted xenograft tumors up to 7 days post‐implantation. Body weights were normalized to the day 0 value. (Aii, Bii) Mean tumor volumes for untreated and NT injury xenograft tumors, along with Cu, OG116‐31 resin, Parylene‐C, Nafion, Pt, Si_3_N_4_, and SiO_2_ implanted xenograft tumors up to 7 days post‐implantation. Tumor volume at each time point was normalized to its day 0 measurement.

The body weights of untreated, biomaterial (SiO_2_, Si_3_N_4_, Parylene‐C, Nafion, OG116‐31 resin, and Pt) and positive control (Cu) implanted mice remained stable throughout the 7‐day experimental period, with no statistically significant changes in body weight observed within any treatment group. Furthermore, no statistically significant differences were identified between the groups at each time point. Mean tumor volumes for untreated, NT and biomaterial implanted tumors all showed growth over the 7 days, with no statistical differences identified between any group at the time points analyzed (Figure 2).

### The effects of biomaterials on tumor necrosis and CA9 staining

**Figure 3:**
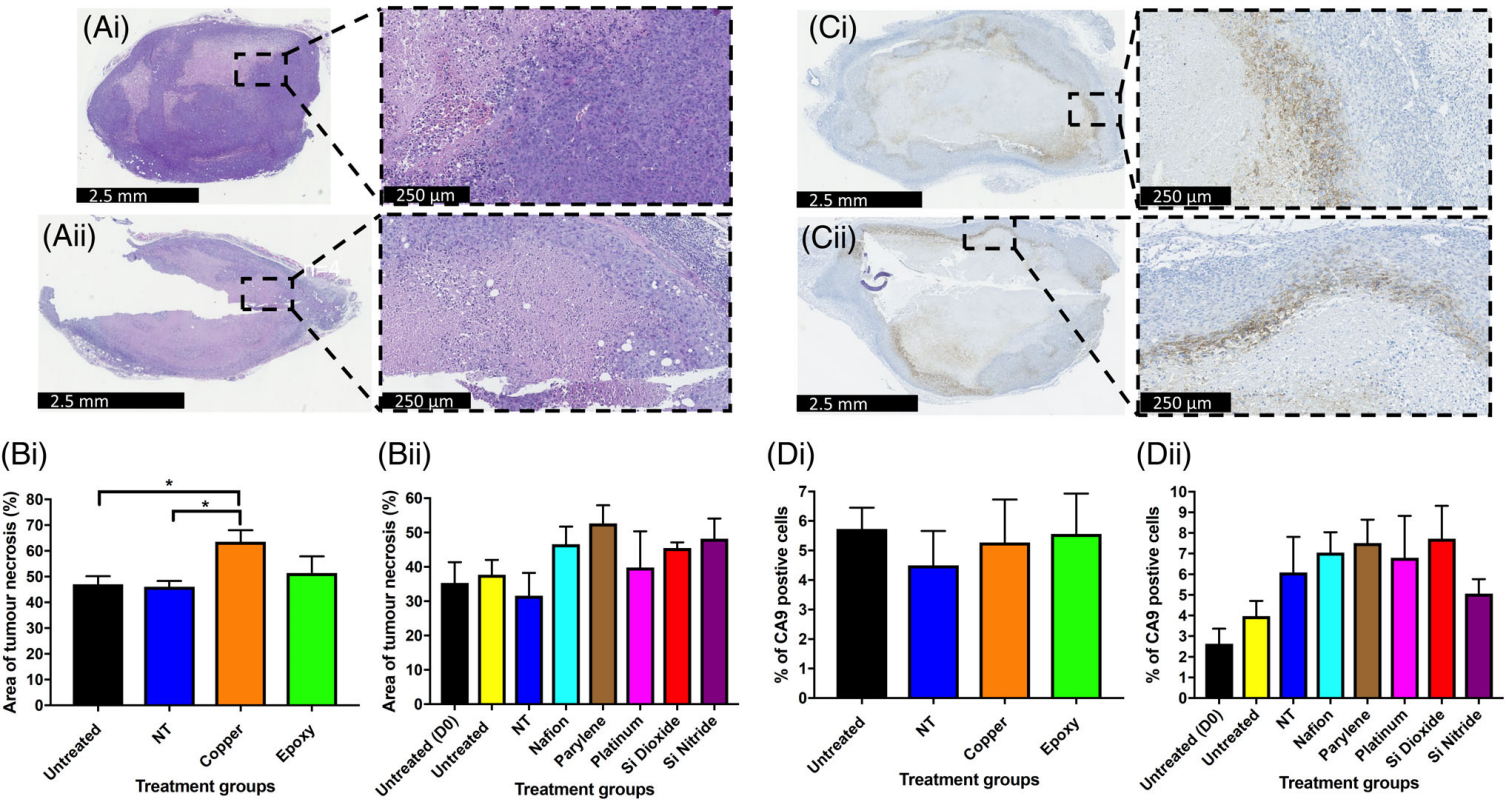
The effects of biomaterials on tumor necrosis and CA9 staining. (Ai, Aii) Representative H&E stained sections from xenograft tumors harvested at day 7. (Ai) Untreated, (Aii) Cu. (Bi, Bii) Percentage area of necrosis for untreated and NT injury xenograft tumors, along with Cu, OG116‐31 resin, Parylene‐C, Nafion, Pt, Si_3_N_4_, and SiO_2_ implanted xenograft tumors up to 7 days post‐implantation. (Ci, Cii) Representative CA9 stained sections from xenograft tumors harvested at day 7. (Ci) Untreated, (Cii) Nafion. (Di, Dii) Percentage of CA9 positive staining cells for untreated and NT injury xenograft tumors, along with Cu, OG116‐31 resin, Parylene‐C, Nafion, Pt, Si_3_N_4_, and SiO_2_ implanted xenograft tumors up to 7 days post‐implantation.

Solid tumors typically have areas of necrosis. At 7 days post‐implantation Cu implanted tumors had a statistically significant increase in the area of necrosis compared to untreated (*p* = 0.0122) and NT (*p* = 0.0361) control groups. No significant difference in the area of necrosis was identified between the remaining biomaterial implanted tumors and control tumors. CA9 is a marker of hypoxia. No significant difference in the percentage of CA9 positive cells was identified between any of the biomaterial implanted tumors and control tumors (Figure 3).

### The effects of biomaterials on tumor proliferation and apoptosis

**Figure 4:**
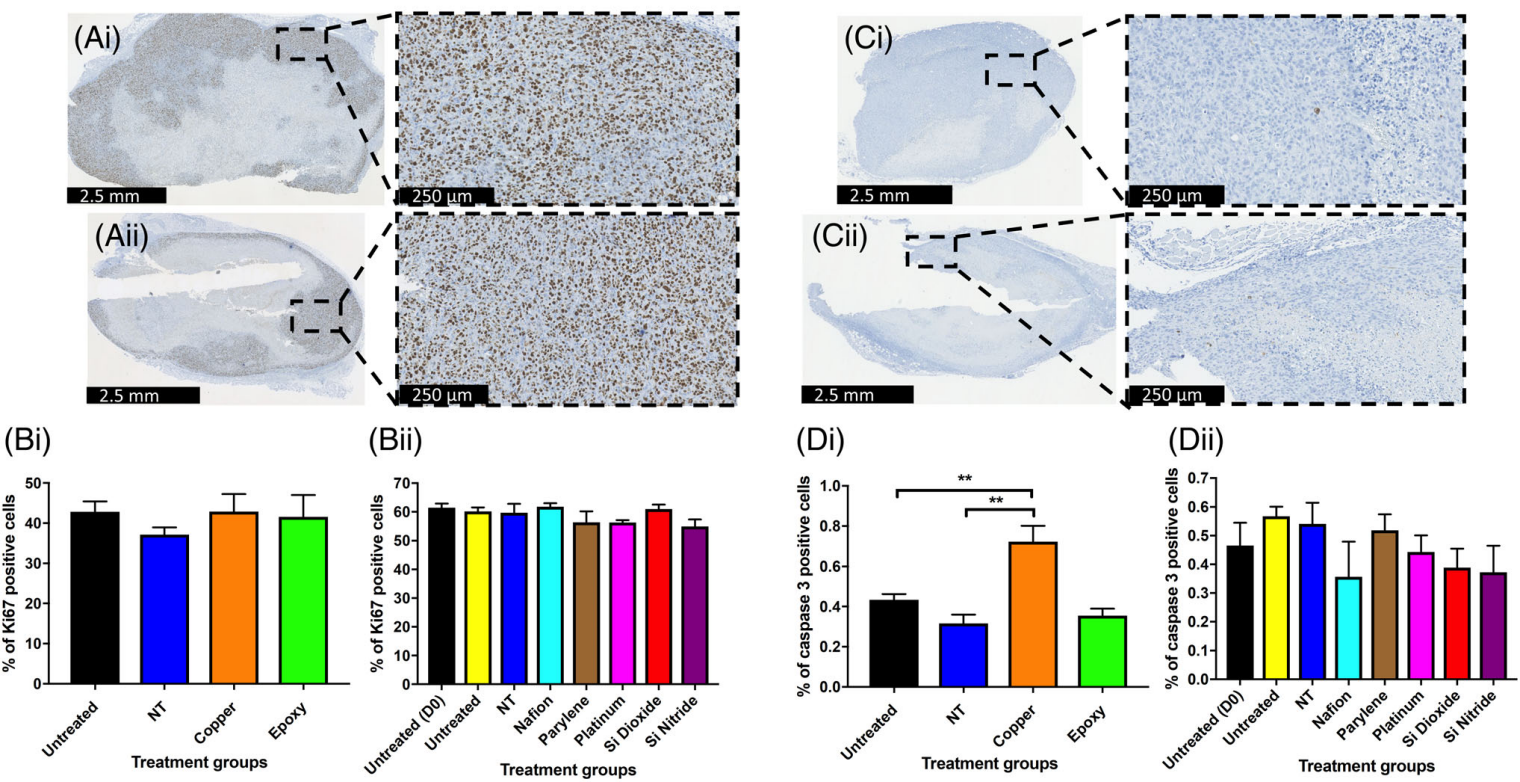
The effects of biomaterials on tumor proliferation and apoptosis. (Ai, Aii) Representative Ki67 stained sections from xenograft tumors harvested at day 7. (Ai) Untreated, (Aii) SiO_2_. (Bi, Bii) Percentage of Ki67 positive staining cells for untreated and NT injury xenograft tumors, along with Cu, OG116‐31 resin, Parylene‐C, Nafion, Pt, Si_3_N_4_, and SiO_2_ implanted xenograft tumors at 7 days post‐implantation. (Ci, Cii) Representative caspase 3 stained sections from xenograft tumors harvested at day 7. (Ci) Untreated, (Cii) Cu. (Di, Dii) Percentage of caspase 3 positive staining cells for untreated and NT injury xenograft tumors, along with Cu, OG116‐31 resin, Parylene‐C, Nafion, Pt, Si_3_N_4_, and SiO_2_ implanted xenograft tumors at 7 days post‐implantation.

Ki67 is a marker of cell proliferation. No significant difference in the percentage of cells staining positive for Ki67 was identified between any of the biomaterial implanted tumors and control tumors. Cleaved caspase 3 is a marker of apoptosis. Only Cu implanted tumors had a statistically significant increase in the percentage of cleaved caspase 3 positive cells compared to untreated (*p* = 0.0033) and NT (*p* = 0.0018) control groups 7 days post‐implantation (Figure 4).

### The effects of biomaterials on neutrophil and macrophage infiltration within tumor tissue

**Figure 5:**
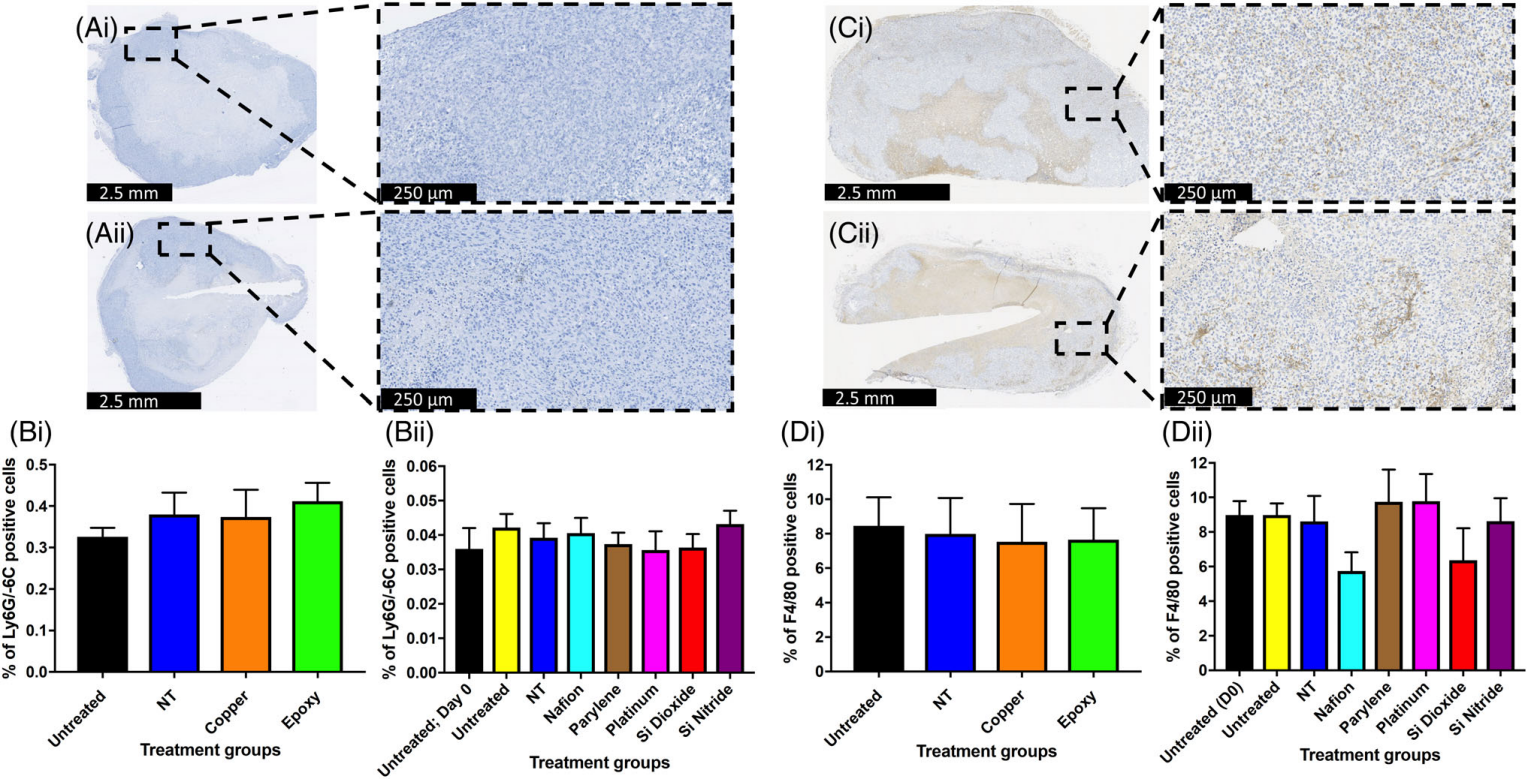
The effects of biomaterials on neutrophil (Ly‐6G/‐6C) and macrophage (F4/80) infiltration within tumor tissue. (Ai, Aii) Representative Ly‐6G/‐6C stained sections from xenograft tumors harvested at day 7. (Ai) Untreated, (Aii) Parylene‐C. (Bi, Bii) Percentage of Ly‐6G/‐6C positive staining cells for untreated and NT injury xenograft tumors, along with Cu, OG116‐31 resin, Parylene‐C, Nafion, Pt, Si_3_N_4_, and SiO_2_ implanted xenograft tumors at 7 days post‐implantation. (Ci, Cii) Representative F4/80 stained sections from xenograft tumors harvested at day 7. (Ci) Untreated, (Cii) Si_3_N_4_. (Di, Dii) Percentage of F4/80 positive staining cells for untreated and NT injury xenograft tumors, along with Cu, OG116‐31 resin, Parylene‐C, Nafion, Pt, Si_3_N_4_, and SiO_2_ implanted xenograft tumors at 7 days post‐implantation.

Ly6G/‐6C is a marker of neutrophils and F4/80 is a marker of macrophages. No significant differences in the percentage of Ly6G/‐6C or F4/80 positive cells were identified between any of the biomaterial implanted tumors and control tumors. Only small numbers of Ly‐6G/‐6C positive cells were identified in any of the tumors (Figure 5).

### The effects of biomaterials on fibroblast infiltration and collagen deposition within tumor tissue

**Figure 6:**
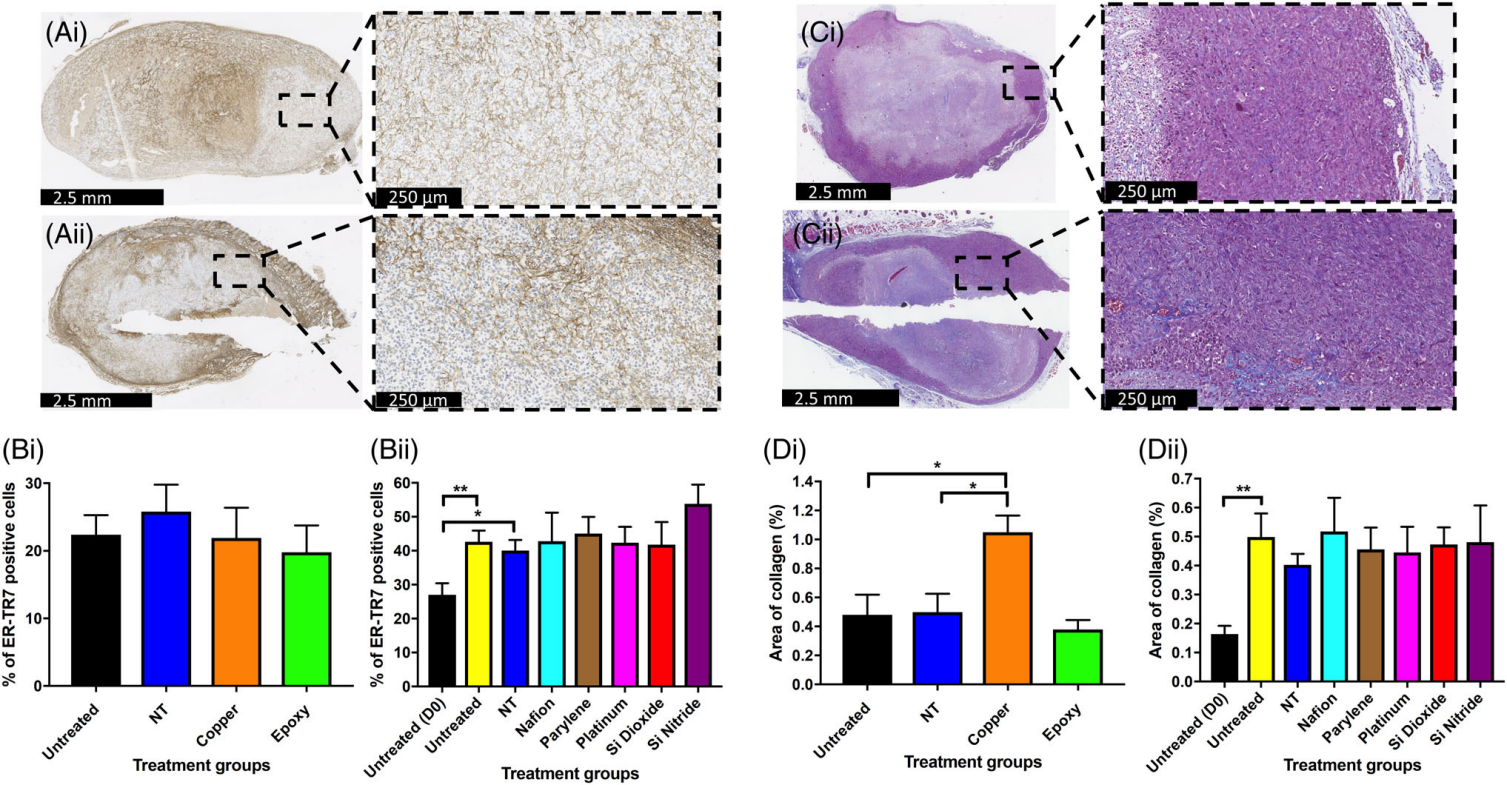
The effects of biomaterials on fibroblast (ER‐TR7) infiltration and collagen deposition within tumor tissue. (Ai–Aii) Representative ER‐TR7 stained sections from xenograft tumors harvested at day 7. (Ai) Untreated, (Aii) Pt. (Bi, Bii) Percentage of ER‐TR7 positive staining cells for untreated and NT injury xenograft tumors, along with Cu, OG116‐31 resin, Parylene‐C, Nafion, Pt, Si_3_N_4_, and SiO_2_ implanted xenograft tumors up to 7 days post‐implantation. (Ci, Cii) Representative Masson's trichrome stained sections from xenograft tumors harvested at day 7. (Ci) Untreated, (Cii) SiO_2_. (Di, Dii) Percentage area of collagen for untreated and NT injury xenograft tumors, along with Cu, OG116‐31 resin, Parylene‐C, Nafion, Pt, Si_3_N_4_, and SiO_2_ implanted xenograft tumors up to 7 days post‐implantation. Data for (Di) graph is expressed as mean ± SEM; according to unpaired two sample *t* test. Data for (Dii) expressed as mean ± SEM; according to one‐way ANOVA followed by Tukey's multiple comparison test.

ER‐TR7 is a marker of fibroblasts. Compared to untreated tumors at day 0, there was a significantly higher percentage of ER‐TR7 positive cells in both untreated (*p* = 0.0073) and NT injury tumors (*p* = 0.0445) at day 7. However, no significant differences in the percentage of ER‐TR7 positive cells were identified between any of the biomaterial implanted tumors and control tumors at 7 days post‐implantation. Untreated tumors at day 7 also had a statistically significant higher percentage area of collagen compared to untreated tumors at day 0 (*p* = 0.0055). Only Cu implanted tumors had a statistically significant increase in the percentage area of collagen compared to untreated (*p* = 0.0135) and NT (*p* = 0.0211) control groups at day 7 post‐implantation (Figure 6).

## DISCUSSION

Interest in the use and development of implantable medical devices has gained momentum in recent years due to their potential roles in precision medicine, and because of technological advances in the development and fabrication of novel biomaterials.^10^ However, any assessment of the ability of an implantable medical device to provide meaningful data must also consider the host response that occurs following implantation. Biocompatibility consists of both biosafety (i.e., appropriate local and systemic host responses with the absence of cytotoxicity, mutagenesis, and/or carcinogenesis) and biofunctionality (i.e., the ability of a device to function with an appropriate host response in a specific application).^9^

To protect patient safety implantable medical devices must conform to standards set by regulatory bodies. The International Standards Agency (ISO) developed and published international standards on the Biological Evaluation of Medical Devices: ISO 10933; this documents a series of standards for biocompatibility evaluation of medical devices prior to clinical testing.^15^, ^45^ Various *in vivo* biocompatibility models including the cage implant system,^16^ the chamber system^17^ and the chorioallantoic membrane (CAM) of a developing chick embryo model^20^ have been described previously; however, these models involve the implantation of materials into normal tissue. If medical devices are developed for implantation into diseased or tumor tissue, then the FBR (as an aspect of biocompatibility) must also be evaluated within these tissues.

Implantable medical devices for cancer treatment have been developed in both clinical and experimental settings.^3^, ^6^ However, only sparse information is available on the FBR within tumor tissue. One historical paper did investigate the FBR after the implantation of cotton thread into rodent tumors, comparing the reaction to that observed in normal tissues. The results showed that the responses seen in tumors were minimal compared to normal tissues, indicating that the FBR may be decreased within tumor tissue.^46^

Here, we assessed the biocompatibility of modern biomaterials under consideration for use in the IMPACT biosensor.^4^ The biocompatibility of these biomaterials was assessed up to 7 days post‐implantation in a human breast cancer xenograft tumor. Histological evaluation was used to assess tumor cell proliferation, apoptosis, hypoxia, and the extent of necrosis. To investigate the FBR, innate immune cell markers encompassing both the acute and beginning of the chronic inflammatory phases (including fibrous encapsulation) were assessed, with Cu used as a positive control. Antibodies selected for use in this study depended on the cell type of interest. Antibodies used to identify immune cells were specifically chosen to detect murine cells, as no human immune cells would be present within this xenograft tumor model; we were therefore assessing the biomaterials effect on the murine immune response rather than a human immune response. This was an important consideration, especially with regards to the F4/80 antibody, as it is solely a murine pan‐macrophage marker. Conversely, antibodies used to investigate ki67 and caspase 3 were required to target antigens present on the MDA‐MB‐231 human breast cancer cells.

Many previous studies have demonstrated the cytotoxic effect of Cu^47^, ^48^, ^49^ mediated through a variety of different mechanisms^50^, ^51^; Cu and Cu ions can participate in the generation of reactive oxygen species (ROS),^52^ which can induce DNA strand breaks and base oxidation, leading to cell death.^53^ Cu^2+^‐induced apoptosis has also been shown to be mediated through ROS, induction of Bax and inactivation of NF‐κB.^54^ Cu induces a stronger inflammatory response in *in vivo* models compared to non‐Cu containing materials, while also leading to larger fibrous capsule development.^55^, ^56^ In our study, Cu implanted xenografts had larger areas of necrosis and an increased percentage of caspase 3 positive cells; the specific mechanism which resulted in Cu producing these changes was not investigated, however, the increased levels of apoptosis observed within the tumors does agree with previously published work.^54^ While the absolute percentage of fibroblasts remained the same in the Cu implanted tumors compared to untreated and NT injury tumors, the percentage area of collagen was increased in Cu implanted tumors, with collagen consisting of 1.05% of the area within the Cu implanted tumors, compared with 0.49% in NT injury tumors and 0.48% in untreated tumors (Figure 6).

The biocompatibility of the biomaterials used in this study has been previously well documented, with results indicating that the materials are well tolerated with a minimal FBR produced when implanted *in vivo*. These results have led to the use of these biomaterials in a variety of medical devices; a detailed summary of these devices can be found in Table 2. However, these previous biocompatibility studies have used models in which materials are implanted into healthy, non‐diseased tissue. Our study was specifically designed to examine the effects of biomaterial implantation within a TME. No systemic toxicities were identified (as indicated by the maintenance of body weight/body condition), and all implanted tumors increased in size over 7 days in accordance with the controls. Assessment of proliferation and apoptosis was performed through Ki67 and caspase 3 staining respectively; no significant differences in cellular proliferation or apoptosis were observed between any of the different treatment groups, indicating that the biomaterials did not affect tumor cell viability. CA9 is a protein induced in hypoxic conditions and is involved in pH regulation^22^; expression of CA9 was investigated as the IMPACT sensor is ultimately designed to monitor tumor O_2_ status. No significant differences in CA9 staining were observed between any of the different treatment groups, indicating that the biomaterials did not affect the O_2_ levels within the tumor. MDA‐MB‐231 xenograft tumors are heterogeneous, containing areas that are normoxic, hypoxic, and necrotic.^103^ The necrosis that develops is a result of the tumor outgrowing its blood supply; although necrotic areas were identified around the implanted materials, the necrotic areas measured were not significantly different from the controls, suggesting no deleterious effects.

**Table 2:** Previously Published Studies Investigating the *In Vivo* and *In Vitro* Biocompatibility Testing That Has Been Conducted on the Biomaterials Investigated in This Article

Biomaterial	*In vitro* Testing	*In vivo* Testing	Implantable Devices	Regulations	TME Biocompatability
**Parylene‐C**	Hemocompatibility testing^57^ Cell culture biocompatability^58^, ^59^, ^60^ Cytotoxicity, hemocompatibility testing^45^	Systemic toxicity, sensitization, intracutaneous reactivity testing^45^ Neural electrodes biocompatability: mice,^59^, ^61^ cat,^62^ monkey,^63^ rat^64^, ^65^, ^66^	Cardiovascular implants^67^	ISO 10993^68^	*In vivo* testing using human breast cancer xenograft tumors up to 7 days post‐biomaterial implantation: No effect on tumor growth rates No significant changes in tumor necrosis, hypoxic cell number (CA9), proliferation (Ki67), apoptosis (caspase 3), immune cell (neutrophil, macrophage), and fibroblast infiltration or collagen deposition
**Platinum**	Cell culture biocompatability^69^		Arterial stents^70^, ^71^ Embolization coils^72^, ^73^ Pacemaker leads, implantable defibrillators, cochlear implants^74^ Brachytherapy devices^75^		
**Silicon Nitride**	Cytotoxicity, hemocompatibility testing^45^ Non‐toxic, encourage cell adhesion, and cellular differentiation^76^, ^77^, ^78^, ^79^, ^80^	Systemic toxicity, sensitization, and intramuscular reactivity testing^45^, ^81^ Fracture repair and evaluation of osteoconduction^76^, ^79^, ^82^, ^83^, ^84^ Cage implant biocompatibility testing^85^, ^86^	Orthopedic and neurosurgical implants^87^, ^88^, ^89^	ISO 10993^77^, ^81^	
**Silicon Dioxide**	Cytotoxicity testing^45^ Biocompatibility and antibacterial testing^80^, ^90^	Intramuscular reactivity testing^45^, ^81^ Nerve electrodes biocompatability: rat^91^ Cage implant biocompatibility testing^85^	Nanomedicine drug delivery systems and diagnostic probes^92^, ^93^	ISO 10993^81^	
**Nafion**	Biocompatibility and antibacterial testing^94^ Non‐cytotoxic and allows cellular differentiation^95^	Subcutaneous, intraperitoneal and intravenous biocompatability: mice,^94^, ^96^ dogs^97^ Intracerebral biocompatability^21^, ^49^, ^98^, ^99^ Nervous system biocompatibility^95^	Electrochemical sensors and electrodes^100^, ^101^		
**EPO‐TEK OG116‐31 resin**	Cytotoxicity, hemocompatibility, genotoxicity testing^45^	Systemic toxicity, sensitization, intracutaneous, and intramuscular reactivity testing^45^	Pacemakers, ophthalmic and neurostimulators, insulin pumps, cochlear implants orthopedic and neurological implants^102^	ISO 10993^102^	

A summary of the results presented in this study is also included.

The FBR generated by an implanted biomaterial has been shown to be affected by the materials modulus, stiffness and mechanics, with materials having lower moduli producing less fibrous capsules than more rigid materials.^104^ Similarly, materials with greater stiffness can cause increased compression, expansion, and subsequent damage to the tissue in which the material is implanted in.^105^ It is therefore thought that softer materials, with properties similar to the implantation tissue, can reduce interfacial strain and improve biocompatibility.^106^ However, the results presented in this study suggest that this may not be a major factor in this tumor model, as no significant differences were seen in the FBR between the hardest (Pt, Youngs modulus 168 GPa) and the softest (Nafion, Youngs modulus 600 MPa) materials tested.

Acute inflammation begins immediately following implantation and can last up to 5 days.^107^ The extent of the acute inflammatory response is related to initial trauma caused by the insertion device, whereas biosensor size has a greater effect on chronic inflammation and the fibrous encapsulation.^108^ Polymorphonuclear leukocytes are the predominant inflammatory cell typically present immediately following and up to 2 days post‐implantation.^11^ This acute inflammatory reaction was assessed through neutrophil numbers using an antibody targeting the Ly6G protein. Ly6G is a glycosylphosphatidylinositol‐anchored protein, also known as the myeloid differentiation antigen Gr1. The antigen is transiently expressed on monocytes in the bone marrow and on eosinophils^109^; however, it is predominantly expressed on neutrophils and is a commonly used marker for murine neutrophils.^110^ In this study, as the percentage of cells that stained positive for Ly6G/‐6C was considerably lower than the percentage obtained for the F4/80 antibody (macrophage marker), it is unlikely that any cross reactivity occurred. Minimal numbers of neutrophils were identified in both the untreated xenografts at day 0 and in all tumors at day 7 post‐implantation. These results suggest that the acute inflammatory response resolved normally in the presence of the biomaterials.

Chronic inflammation develops when the inflammatory stimulus (biomaterial) remains at the implantation site and can last up to 3 weeks following the resolution of acute inflammation; it is marked by the presence of monocytes, macrophages, and fibroblasts, with the development of neovascularization and the production of granulation and fibrous tissue.^11^ Macrophages are the predominant cell type present that drive the continuing immune response.^11^, ^111^ Macrophages can phagocytose particles up to 5 μm in size, however, larger particles (as in the case of biomaterials) will cause macrophages to coalesce forming foreign body giant cells (FBGCs).^112^ Alternatively activated macrophages can produce profibrogenic factors leading to enhanced fibrogenesis by fibroblasts.^113^ Biomaterial adherent macrophages can therefore secrete proteins that modulate fibrosis, causing the deposition of a collagenous, and vascular fibrous capsule around the biosensor. This fibrous capsule is the end stage of the FBR^56^ and can be 50–200 μm thick.^9^ In conjunction with FBGC on the implant surface, the fibrous capsule creates a barrier confining the implant, preventing it from interacting with the surrounding tissue; this can contribute to a loss of device function. The potential for this was investigated through macrophage and fibroblast staining, while also assessing Masson's trichrome for collagen deposition.

Although it is possible to differentiate between tissue‐resident macrophages and tumor‐associated macrophages (TAMs) in human and mouse tumors, it is challenging to do so.^114^ TAMs have been shown to promote tumor cell migration, invasion, metastasis and the induction of angiogenesis, all of which are important processes associated with malignant progression.^114^ Macrophage identification was performed using an antibody reactive to murine F4/80, a protein that has been widely used as a pan‐macrophage marker^115^, ^116^; as such, we did not distinguish between the different macrophage phenotypes. The observation of a similar total number of macrophages in both the control and implanted tumors indicates that the materials did not lead to the recruitment of additional macrophages. Ongoing tumor growth may have led to the statistically significant increase in fibroblast number and collagen percentage identified between day 0 untreated and day 7 untreated/NT injury tumors.

Since the initial observation by Rygaard and Povlsen^117^ that human tumors could be successfully grown in athymic nude mice, various mouse models (including NOD SCID and CD‐1 nude mice) have been extensively used to study the growth characteristics, metastatic potential, morphology, and function of numerous xenograft human neoplasms.^118^, ^119^ CD‐1 nude mice were used in this study due to the relative ease, reproducibility, and speed of producing human xenograft tumors for implantation within this model. Although athymic nude mice are deficient in thymus derived T lymphocytes, they are not fully immunodeficient. Nude mice can still produce diminished numbers of T lymphocytes via thymus‐independent pathways and can therefore mediate some degree of T cell dependent immunity.^120^ They also show a near‐normal response to T‐cell‐independent antigens and have high titres of natural antibodies that can react with tumor cells.^121^ Tumoricidal macrophages can also be isolated from nude mice, and their activity can be enhanced after *in vivo* stimulation with bacterial adjuvants.^122^ Studies have also shown that nude mice consistently exhibit a high level of natural killer (NK) cell activity.^123^ These studies provide evidence of the ability of these mice to mount both an adapted and innate immune response.

The use of T cell deficient mice for investigation of the FBR to implanted materials has previously been investigated^124^; this is an important consideration as T cells are present briefly in the chronic phase of inflammation, and *in vivo* lymphocyte/macrophage co‐cultures demonstrate that lymphocytes can increase macrophage adhesion to biomaterial surfaces and enhance the formation of FBGC; furthermore, the presence of macrophages can stimulate lymphocytes to proliferate.^125^ However, the specific role for macrophage/lymphocyte interactions has yet to be identified, and previous studies have shown that an appropriate FBR can occur in the absence of T cells.^124^ This study used BALB/c nude mice, which are T‐cell deficient through the lack of a thymus. Although these T‐cell‐deficient mice had lower total leukocyte concentrations at the biomaterial implant site, FBGC morphology, and number were comparable to the BALB/c mice, suggesting that pathways independent of thymus‐matured T lymphocytes can still lead to a normal FBR following biomaterial implantation. Using non‐immunosuppressed models, such as carcinogen‐induced tumors, would create tumors in the presence of an intact immune system; however, the model would not then have the benefit of using xenografted human tumor cells and therefore would not assess the effects of the implanted biomaterials on a human cancer.

**Figure 7:**
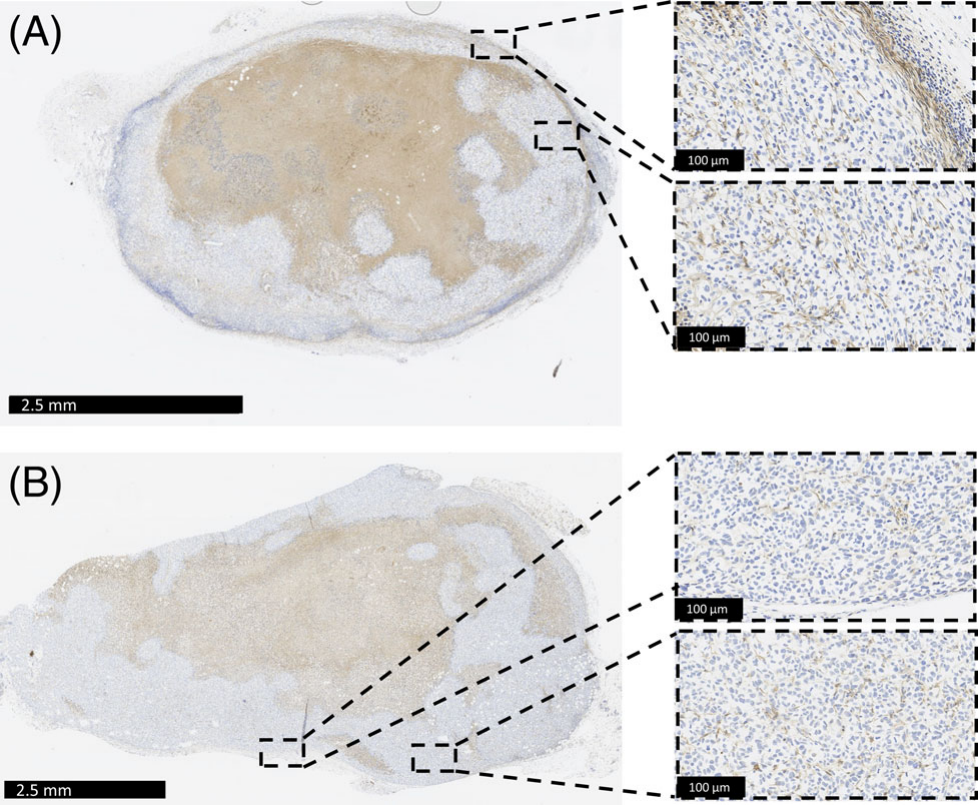
Macrophage (F4/80) distribution types within untreated xenograft tumors. (A) Macrophages are identified both at the periphery of the tumor and within the tumor tissue. (B) Macrophages are uniformly distributed within the tumor tissue.

The pathological equivalence of nude mice xeno‐transplanted human tumors and naturally occurring tumors in humans needs to be considered, as there is evidence that changes in tumor characteristics (histological classification and proliferation rates) can occur after transplantation.^118^, ^119^ The murine host response to the presence of human cancer cells/tissue is another factor that needs to be considered when using CD‐1 nude mice. In human cancers macrophages are uniformly distributed throughout tumor tissue.^126^ While this distribution was seen in some of the tumors analyzed in this study, a number of tumors had macrophages predominately localized toward the outside of the cancer tissue (Figure 7). This type of macrophage positioning has been documented in previous studies and may be explained by the macrophages associating with the formation of a fibrous capsule surrounding the tumor itself.^126^ Whether or not the localization of immune cells within/around a human xenograft tumor would affect the FBR to an implanted material is unknown; however, as we saw an equal mixture of macrophage distributions in control and implanted tumors, the effect is likely to be minimal.

Differences in the structure and function of mice and human immune systems also need to be considered when evaluating data from mice studies and extrapolating it to humans. A comprehensive comparative study/review investigated differences in the immune system in a range of species, including CD‐1 mice and humans.^127^ One potentially relevant finding to our study was that circulating leukocyte populations have marked species differences. They stated that the circulating leukocyte profile in whole blood for humans is considered to be neutrophilic, having approximately 50–70% neutrophils (3500–7000 cells/μL) and 20–40% lymphocytes (1400–4000 cells/μL), whereas in CD‐1 mice there is a greater percentage of circulating lymphocytes; 15–20% neutrophils (300–2000 cells/μL) and 50–70% lymphocytes (1000–7000 cells/μL). In addition to differences in the absolute numbers of circulating leukocytes, there are also variations in neutrophil function and activity between the species. Factors involved in leukocyte recruitment, such as chemoattractive signals and rolling adhesion, are known to act though different mechanisms in both humans and mice.^128^ Composition of neutrophil granules also vary between mouse and human neutrophils; for example, mice lack defensins, which can determine their antimicrobial mode of action.^128^ These differences in circulating leukocyte number and function could potentially result in an alteration in the initiation and progression of a FBR between the two species, as neutrophils are required in the initial acute inflammatory phase^11^ and lymphocytes are transiently present in the chronic phase of the FBR.^125^

Nitric oxide (NO) is known to have a role in the immune system, influencing the activity of many different immune cell types.^129^ NO has also been shown to have an effect on the FBR, with increased NO levels leading to a reduced FBR.^130^ While studies have shown that mouse macrophages have the ability to produce NO, there are differing reports on the ability of human macrophages to generate NO^128^; variations in NO production may therefore lead to a difference in the FBR between the two species. Divergences in the immune systems of mice and humans, highlighted here using neutrophils, lymphocytes, and macrophages as examples, emphasize the need for caution when attempting to directly translate data from murine disease models to human pathologies.

Endotoxin testing was not carried out on the materials used in this study. This may have been a confounding factor if any of the materials had produced a significant inflammatory response in implanted tumors compared with the controls. The percutaneous method employed, either to implant the materials within the tumors or cause a NT injury in control tumors, also had the potential to introduce endogenous cutaneous bacterial or endotoxins inside the tumor. However, protocols to minimize these issues were used, such as aseptic skin preparation, wire sterilization, along with minimal anesthesia and surgery time.^131^ No visible signs of inflammation occurred at the implant site, all mice remained healthy for the duration of the experiment, and the control and implanted tumors did not differ in terms of inflammation; these results suggest that the potential confounding factors noted above were not an issue. A previous study used a similar percutaneous method to evaluate the FBR in mice. Nylon mesh was loaded into a 16‐gauge needle, which was inserted subcutaneously, and a sterile syringe plunger was used to push the implant into the subcutaneous tissue.^116^ Sham mice also underwent a NT injury similar to our procedure. The advantages of our model over this study is that a much smaller gauge of material or needle (for NT injury) was used; this is an important consideration in any FBR model, as the extent of the acute inflammatory response is related to the initial trauma caused by the insertion device.^108^

Biomaterial effects were assessed for 7 days post‐implantation; this time period includes the acute inflammatory response and the beginnings of the chronic inflammatory response. For full evaluation of the chronic inflammatory response, materials would need to be implanted for up to 3 weeks, a time frame which would be difficult to achieve using a murine model. However, Parylene‐C implantation was conducted up to 14 days post‐implantation (data not shown), which produced results similar to those described here.

## CONCLUSION

This is the first, crucial step in determining if SiO_2_, Si_3_N_4_, Parylene‐C, Nafion, OG116‐31 resin, and Pt are suitable materials for implantable medical devices placed within a solid tumor. Our results suggest that the materials caused no deleterious effects on the tumor and do not trigger a significant FBR. However, it should be noted that additional biocompatibility testing is required to fully investigate the FBR. Experiments using functional biosensors, implanted into a tumor in immunocompetent large animal models for longer periods, would provide further confidence in the biocompatibility of materials included in the sensors produced by the IMPACT project.

## CONFLICT OF INTEREST

There are no conflict of interest.
